# Application of telemedicine technology for cardiovascular diseases management during the COVID-19 pandemic: a scoping review

**DOI:** 10.3389/fcvm.2024.1397566

**Published:** 2024-08-12

**Authors:** Hassan Asadi, Esmaeel Toni, Haleh Ayatollahi

**Affiliations:** ^1^ Department of Health Information Management, School of Health Management and Information Sciences, Iran University of Medical Sciences, Tehran, Iran; ^2^ Student Research Committee, Iran University of Medical Sciences, Tehran, Iran; ^3^ Health Management and Economics Research Center, Health Management Research Institute, Iran University of Medical Sciences, Tehran, Iran

**Keywords:** telemedicine, telehealth, disease management, cardiovascular diseases, COVID-19

## Abstract

**Background:**

Recently, the use of telemedicine technology has increased due to the Covid-19 pandemic. Cardiovascular diseases management is one of the areas that has benefited from using this technology. This study aimed to identify the applications of telemedicine for cardiovascular diseases management during the Covid-19 pandemic.

**Methods:**

This scoping study was conducted in 2023. Various databases, including PubMed, Web of Science, Scopus, the Cochrane Library, Ovid, CINAHL, ProQuest, and IEEE Xplore along with Google Scholar were searched and all related quantitative, qualitative, and mixed-method studies published in English between 2020 and 2022 were included. Finally, the required data were extracted, and the findings were reported narratively.

**Results:**

A total of 17 articles were included in this study. The results showed that teleconsultation via telephone and videoconferencing were the most common used technologies. Telemedicine helped to improve clinical impacts such as patient health status and quality of care, and reduced hospitalizations and re-admission rates compared to in-person visits. The non-clinical impact included reducing waiting time, in-person visits, and healthcare costs during the Covid-19 pandemic.

**Conclusion:**

The Covid-19 pandemic has led to an increased use of telemedicine technology, especially for patients with cardiovascular diseases. As teleconsultation and telemonitoring are useful for cardiovascular diseases management and regular examinations, future research should investigate how these technologies can be improved and used for a wider population.

## 1 Introduction

Cardiovascular diseases are one of the main causes of worldwide death (1, 2). Nearly half of all deaths in high-income countries and about 28% of deaths in low- and middle-income countries are caused by cardiovascular diseases (3, 4). According to the literature, approximately 23.6 million people will die due to cardiovascular diseases by 2030 (2, 5–7) Therefore, many interventions have been carried out to reduce the burden of diseases and the number of deaths, to provide the necessary training for better prevention and treatment, and to use new technologies such as telemedicine for better patient-provider interactions (6–8).

In late 2019, when the world faced with the Covid-19 pandemic (9), patients with cardiovascular diseases were more at risk of adverse consequences caused by infections and complications of the Covid-19 disease (10, 11). Moreover, the pandemic caused significant cardiometabolic risk factors that could worsen the prognosis for cardiovascular patients. The cytokine storm and visceral obesity were among these risk factors. The cytokine storm, a hyperactive immune response triggered by Covid-19, coud lead to severe inflammation and multi-organ failure, particularly affecting the cardiovascular system. Visceral obesity, a condition characterized by excess fat around the internal organs, was identified as a major risk factor for severe Covid-19 outcomes, including heart failure and atherosclerosis (12).

Therefore, the application of telemedicine technology increased, as this technology helped with reducing the spread of the disease by decreasing patient attendance in the crowded places such as hospitals (13). In addition, the technology was easy to use and provided the patients with low-cost services (14).

The use of this technology for cardiovascular patients during the Covid-19 pandemic minimized the risk of disease transmission at primary care, provided optimal treatment, and prevented the deterioration of the clinical conditions. It also led to early signs monitoring of heart failure and reduced unnecessary hospitalizations (15–17).

Molinari et al.'s study showed that the use of telecardiography technology for cardiovascular patient management led to reduction in non-emergency and emergency visits by half and a quarter, respectively (18). In another study, Ajibade et al. found that the use of telemedicine services can be effective in pre-and post-operative care and for treating patients with cardiovascular diseases during the Covid-19 pandemic (9).

Although, several review studies have been conducted on the use of telemedicine technology for treating patients with cardiovascular diseases during the Covid-19 pandemic (9, 19–22), few studies have investigated different types of technologies, services, and clinical and non-clinical impact of telemedicine on cardiovascular diseases management during the Covid-19 pandemic. Therefore, the present study aimed to identify the applications of telemedicine for cardiovascular diseases management during the Covid-19 pandemic.

## 2 Methods

In this scoping review, Arksey and O'Malley's framework was used (23). A scoping review can help to provide a comprehensive overview of the research topic and to combine the results of different study types. In addition, conducting a scoping review allows for faster and more cost-effective resolution of the gaps in the existing knowledge and the extraction of essential data elements from different studies.

### 2.1 Stage 1: identify the research question

The starting point for developing a search strategy in review studies is identifying the research question. Therefore, the research question for the present study was generated as follows:

What were the applications of telemedicine technology for cardiovascular diseases management during the COVID-19 pandemic?

### 2.2 Stage 2: identifying of relevant studies

To identify relevant studies, search strategies were developed and eight databases including PubMed, Web of Science, Scopus, the Cochrane Library, Ovid, IEEE Xplore, ProQuest, CINAHL, and the Google Scholar search engine were searched for studies that were published in English between 1st June 2020 and 31th December 2022. The search strategy consisted of the three main terms “Covid-19”, “telemedicine”, and “cardiovascular diseases” which were combined using AND/OR logical operators (Appendix I). To increase reliability, the reference list of the included studies and their citations were also examined.

### 2.3 Stage 3: selection of the studies

To select the relevant studies, specific inclusion and exclusion criteria were set. All original articles published in English that used different types of research methodologies including quantitative, qualitative, and mixed-methods approaches to evaluate the use of telemedicine interventions for cardiovascular diseases management during the Covid-19 pandemic were included in this study.

Review articles, letters to the editor, protocols, and studies that did not reported the use of telemedicine technology for cardiovascular diseases management as an intervention were excluded from the current study. In addition, studies that were not published in English, did not report clinical or non-clinical impacts, and their full-texts were not available were all excluded from the review.

### 2.4 Stage 4: data charting

After searching articles, the retrieved studies were entered into the EndNote software. Then, duplicates were removed, and the remaining articles were evaluated based on the relevance of their titles and abstracts to the objective of the present study. Subsequently, the full texts of the eligible studies was obtained and examined. Both authors (HA and ET) independently screened the articles and resolved any disagreements through discussion. Any disagreements were discussed and solved with the help of the third author (HA). The screening process of articles was undertaken based on the Preferred Reporting Items for Systematic reviews and Meta-Analyses extension for Scoping Reviews (24).

Having read the full texts of the included studies, the required data were extracted using a data extraction form. The data included the name/s of the author/s, year of publication, country, study objective, research methodology, type of telemedicine services, type of telemedicine technology, the impact of technology, and a summary of the main results.

### 2.5 Stage 5: collecting, summarizing, and reporting the results

The extracted data were tabulated, summarized, and reported narratively. Results were reported by categorizing studies according to the type of telemedicine services, type of technology, and clinical and non-clinical impact.

## 3 Results

**Figure 1:**
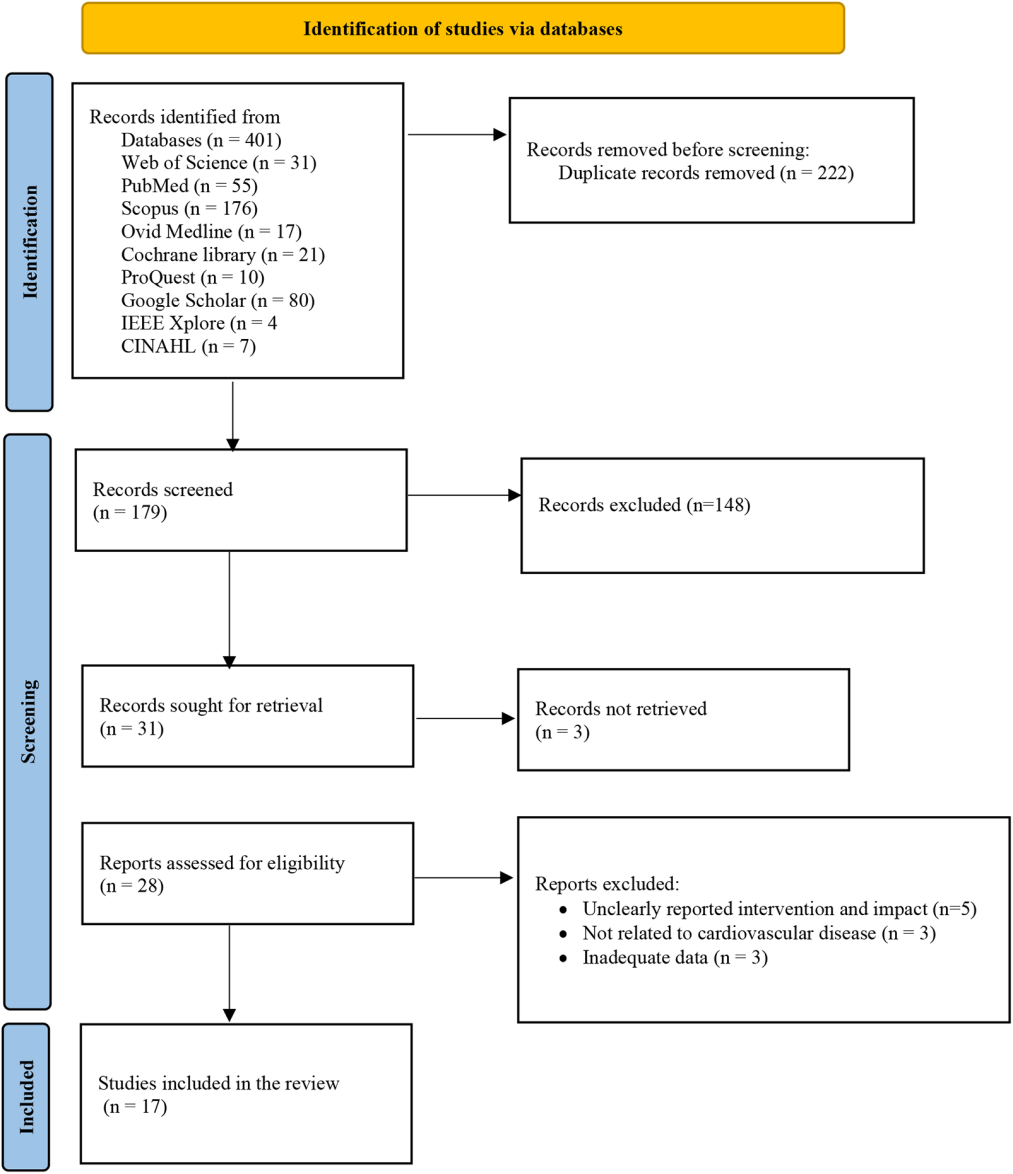
Selecting papers based on the PRISMA-ScR checklist (24).

Initially, 401 papers were retrieved. After removing duplicated and completing the screening process, 17 articles met the inclusion criteria and were selected for further review (7, 15, 16, 18, 25–37). Figure 1 shows the process of selecting articles based on the PRISMA diagram.

### 3.1 Characteristics of the selected studies

Most of the included studies were conducted in the USA (*n* = 5) (25, 26, 29, 30, 32). Other studies were conducted in Italy (*n* = 3) (18, 28, 36), India (*n* = 2) (7, 33), Australia (*n* = 1) (35), Czech Republic (*n* = 1) (16), England (*n* = 1) (27), Portugal (*n* = 1) (15), Thailand (*n* = 1) (34), China (*n* = 1) (37) and Egypt (*n* = 1) (31). Four studies were published in 2022, and 13 related studies were published in 2021. Most of the studies used quantitative methods and included before-after studies (*n* = 6) (7, 16, 18, 28, 32, 34), retrospective cohort studies (*n* = 2) (29, 30), analytic cross-sectional studies (*n* = 3) (26, 35, 36), descriptive cross-sectional research (*n* = 2) (33, 37), randomized controlled trial studies (*n* = 2) (25, 31), and a prospective cohort study (*n* = 1) (15). Only one study used a mixed-methods exploratory design (27). Table 1 shows more details about the included studies.

**Table 1:** Summary of the studies.

No.	Authors/year	Country	Objective	Research methodology	Type of telemedicine services	Type of telemedicine technology	The impact of technology	Results
1	Kalvani et al./2022 (29)	USA	To investigating visit volumes and telemedicine utilization across cardiology subspecialties at an academic cardiovascular center during the initial twelve months of the Covid-19 pandemic.	Quantitative (retrospective cohort study)	Televisit	Video conferencing (Cardio Click platform)	Clinical impact: – Reducing systolic blood pressure -Reducing diastolic blood pressure – Reducing low-density lipoprotein cholesterol – Improving Statin use Non-clinical impact: – Reducing waiting time – Reducing visiting time – Improving documentation quality	Televisit significantly reduced patient waiting time (*P* < 0.01), and physicians were more likely to complete patients’ medical records than in-person visits. (*P* = 0.0016)
2	Brewer et al./2022 (25)	USA	To investigating a mobile phone health intervention as part of the program to improve and strengthen the overall health of African Americans to promote cardiovascular health during the Covid-19 pandemic.	Quantitative (randomized controlled trial)	Telemonitoring	Mobile application	Clinical impact: – Improving physical activity -Improving nutritional well-being	The use of mobile application was generally high, and it was also regarded as a reliable resource for patients seeking to maintain a healthy lifestyle.
3	Mohan et al./2022 (33)	India	To identify the feasibility of using teleconsultation to manage cardiovascular patients during the Covid-19 pandemic and its impact on patient satisfaction	Quantitative (cross-sectional descriptive study)	Teleconsultation	Telephone	Clinical impact: – Reducing hospital and medical center referrals Non-clinical impact: – Increasing patients’, physicians’ and nurses’ satisfaction	Patients were more satisfied with physicians’ telephone consultation approaches than consuting with nurses. The use of teleconsultation services resulted in a small number of patients (6.3%) being referred to other centers for additional services, with only 8.4% of these patients requiring hospitalization.
4	Liang et al./2022 (32)	USA	To investigating the effects of telegenetics on clinical cardiogenetic practices in New York City during the Covid-19 pandemic.	Quantitative (before and after study)	Telegenetics	Telephone and video conferencing	Clinical impact: – Increasing the number of televisits	The proportion of telegenetics visits increased from 6% in 2019 to 80% in 2020. The number of patients seeking genetic counseling increased from 18% in 2019 to 34% in 2020. By 2020, genetic counseling had expanded far beyond New York State, reaching 11 states.
5	Kalwani et al./2021 (30)	USA	To investigate the operational impact of a teleconsultation program for cardiovascular disease in an academic medical center during the Covid-19 pandemic	Quantitative (retrospective cohort study)	Teleconsultation	Video conferencing (Cardio Click platform)	Non-clinical impact: – Reducing physician appointment time-Increasing the number of physician visits	The use of teleconsultation resulted in a mean time reduction of 48 min per patient compared to in-person visits. Teleconsultations were more likely to result in a successful visit than in-person visits. (*P* < 0.001) Teleconsultation utilization resulted in an increased number of patient visits compared to in-person visits.
6	Friedman et al./2021 (26)	USA	To evaluate a virtual cardiovascular care program for reducing readmissions of patients with heart failure after one month of hospitalization during the Covid-19 pandemic	Quantitative (analytical cross-sectional study)	Teleconsultation	Video conferencing, telephone consultation and access to electronic medical records (Heart Beat Health platform)	Clinical impact: – Reducing patients’ re-admission – Non-clinical impact – Saving cost	The use of telemedicine services led to a reduction in patient readmissions by 9% and cost savings of $860 per patient compared to in-person visits.
7	Batalik et al./2021 (16)	Czech Republic	To investigate the effects of a home-based telecardiac rehabilitation program and the walking exercise test on coronary heart disease patients during the Covid-19 pandemic	Quantitative (before and after study)	Telerehabilitation	Wireless sensors and web-based application	Clinical impact: – Improving cardiorespiratory fitness – Reducing walking test time	Telerehabilitation improved cardiorespiratory fitness significantly (*P* < 0.001), resulting in an 8% reduction in walking test time.
8	Farit et al./2021 (27)	United Kingdom	To investigate changes in patient activation measures among patients participating in cardiac rehabilitation via the Active + m program during the covid-19 pandemic.	Mixed-methods study (sequential exploratory design)	Telemonitoring	Wireless sensor and mobile application (using Active + Me platform)	Clinical impact: – Improving patient activity, systolic blood pressure, waist circumference, physical activity, and mental well-being – Improving safety of patients against the Covid-19 pandemic	Participants agreed that telemonitoring would reduce the limitations of in-person visits and improve self-confidence, spontaneity in goal setting, self-monitoring, and self-efficacy. Telemonitoring significantly improved patient activity measures (*P* = 0.03), systolic blood pressure (*P* = 0.02), waist circumference (*P* = 0.02), and physical activity (*P* = 0.001).
9	Sachdeva et al./2021 (7)	India	To investigate the impact of teleconsultation services in pediatric cardiac care delivery in the Covid-19 era	Quantitative (before and after study)	Teleconsultation	Telephone	Clinical impact: – Reducing hospitalizations, and emergency surgeries Non-clinical impact – Reducing patients’ physical presence duration	Compared to before the COVID-19 pandemic, teleconsultation replaced telephone consultations with outpatient services and reduced inpatient admissions, operations, and emergency operations by 66%, 88%, and 40%.
10	Molinari et al./2021 (18)	Italy	To assess the impact of Covid-19 on telemedical cardiovascular diseases management in three medical service centers	Quantitative (before and after study)	Televisit	Teleelectrocardiogram	Clinical impact: – Reducing electrocardiogram-based disease diagnosis duration, non-emergency and emergency calls Non-clinical impact: – Reducing in-person visits duration	Televisit significantly reduced the duration of disease diagnosis on electrocardiogram (*P* < 0.001) and lowered non-emergency and emergency calls by half and a quarter, respectively.
11	Afonso et al./2021 (15)	Portugal	To investigating the effects of telemedicine on management and reducing the risk of Covid-19 infection in patients with heart failure during the Covid-19 pandemic	Quantitative (prospective cohort study)	Teleconsultation	Telephone, text message, and e-mail	Clinical impact: Reducing number of hospitalization and face-to-face visits	The use of telemedicine services resulted in a significant difference in the rate of hospital admissions (*P* = 0.83) and visits (*P* = 0.28) compared to before the Covid-19 pandemic
12	Powananth et al./2021 (34)	Thailand	To investigate the impact of the COVID-19 pandemic and the Tele-HF Clinic (Tele-HFC) program on cardiovascular death, heart failure (HF) re-hospitalization, and heart transplantation rates in a cohort of ambulatory HF patients during and after the peak of the pandemic.	Quantitative (before and after study)	Teleclinic	Telephone and video conferencing	Clinical impact: Reducing heart transplant surgeries Reducing drugs dosage and readmissions rates	Use of the teleclinic significantly reduced the rate of heart transplant surgery (*P* = 0.007), readmission (*P* = 0.006), and improved heart failure (*P* = 0.002) compared to before the Covid-19 pandemic. Long-term teleclinic use significantly reduced the dosage of angiotensin-converting enzyme inhibitors (*P* = 0.006), beta-blockers (*P* = 0.001), furosemide (*P* = 0.002), and tolvaptan (*P* = 0.002).
13	Russo et al./2021 (36)	Italy	To evaluation medical interventions after teleconsultation for ambulatory management of patients with cardiovascular diseases during the covid-19 pandemic	Quantitative (analytical cross-sectional study)	Teleconsultation	Telephone	Clinical impact: Monitoring patient health condition	Patients with a higher risk of cardiovascular diseases (*P* < 0.003), dyslipidemia (*P* < 0.001), and coronary artery disease (*P* < 0.0008) were more likely to use teleconsultation services than other patients.
14	Rowe et al./2021 (35)	Australia	To identify characteristics contributing to choosing telephone versus video consultation and assess patient outcomes between telehealth modalities.	Quantitative (analytical cross-sectional study)	Teleconsultation	Telephone or video conferencing	Clinical impact: Increasing follow-up visits	Video conferencing resulted in increasing the number of appointments for follow-up compared to the telephone consultation (*P* = 0.015).
15	Grandinetti et al/2021 (28)	Italy	To describe the experience of implementing a telemedicine service for adults with congenital heart disease (ACHD) during the COVID-19 pandemic	Quantitative (before and after study)	Teleconsultation	Telephone	Clinical impact: – Increasing the number of visits – Reducing heart palpitations and lower extremity edema – Improving respiratory symptoms	Teleconsultation resulted in a significant reduction in palpitations (*P* < 0.01), lower extremity edema (*P* < 0.01), and improvement in respiratory symptoms (*P* < 0.01) compared to in-person treatment conditions before the Covid-19 pandemic.
16	Kamel et al./2021 (31)	Egypt	To investigating the value of telemedicine in the Covid-19 pandemic for the short-term medical management of acute myocardial infarction following primary percutaneous coronary intervention	Quantitative (randomized controlled trial)	Teleconsultation	Video conferencing (Zoom platform)	Clinical impact: – Improving cardiac rehabilitation – Increasing adherence to smoking cessation Non-clinical impact: – Improving patient satisfaction – Saving time and travel cost – Reducing physician's workload.	Teleconsultation significantly improved medication adherence (*P* < 0.02), smoking cessation (*P* = 0.001), and cardiac rehabilitation (*P* = 0.001). About 87% of patients were satisfied with the telemedicine services, and many patients were satisfied with the virtual visits even after they were discharged. About 61% of patients believed that the teleconsultation was as effective as visiting the clinic.
17	Zhang et al./2021 (37)	China	To investigate the impact of WeChat-based telehealth services on the preoperative follow-up of infants with congenital heart disease during the Covid-19 pandemic.	Quantitative (descriptive cross-sectional study)	Teleconsultation	Messenger (WeChat)	Clinical impact: – Facilitating early diagnosis of respiratory infections – Improving nutritional wellbeing, shortness of breath, lack of weight gain, and nutritional counseling – Reducing patient caregivers’ anxiety	The teleconsultation was used to diagnose respiratory infections, nutritional problems, shortness of breath, and weight gain, after which nutritional recommendations and individualized follow-up were provided to the children's parents. Teleconsultation reduced anxiety and depression in patients’ parents.

### 3.2 Types of telemedicine services

The results showed that teleconsultation (*n* = 10) (7, 15, 26, 28–30, 33, 35–37), televisit (*n* = 2) (18, 29), telemonitoring (*n* = 2) (25, 27, 32), telegenetics (*n* = 1) (32), telerehabilitation (*n* = 1) (16), and teleclinic (*n* = 1) (34) services were used to manage cardiovascular diseases during the Covid-19 pandemic.

Teleconsultation was used as the most common telemedicine services for a variety of cardiovascular diseases, such as heart failure (7, 15, 26, 30, 33, 36), congenital heart disease (28, 37), acute myocardial infarction (31) and outpatient cardiac patients with emergencies (35) during the Covid-19 pandemic.

### 3.3 Types of telemedicine technologies

According to the results, telephone consultations (*n* = 9) (7, 15, 26, 28, 32–36), videoconferencing (*n* = 7) (26, 29–31, 34, 35) via platforms such as CardioClick (29, 30), Heartbeat Health (26), and Zoom (31), mobile applications (*n* = 3) (16, 25, 27), wireless sensors (*n* = 2) (16, 27), tele-electrocardiogram (*n* = 1) (18), messenger (*n* = 1) (37), text messaging (*n* = 1) (15), and email (*n* = 1) (15) were used to manage patients with cardiovascular diseases during the Covid-19 pandemic.

These technologies had several advantages. Mobile applications helped to improve patient health status (25, 27) and cardiorespiratory fitness (16). Videoconferencing was effective in reducing time for being visited (29) and number of re-hospitalizations (26). Telephone-based consultations facilitated hospitalization (33, 34), caring for heart transplantation (34), and providing nursing services (36).

### 3.4 The impact of using telemedicine services

The impact of using telemedicine services on cardiovascular diseases management were divided into two categories: clinical impact (e.g., reducing re-admissions rates, improving patient condition, etc.), and non-clinical impact (e.g., reducing waiting times and increasing satisfaction, awareness, etc.). The details of these categories are presented in the below sections.

#### 3.4.1 Clinical impact

Patients with cardiovascular diseases benefited from using telemedicine services during the Covid-19 pandemic. It improved clinical symptoms (27, 29), low-density lipoprotein cholesterol (29), shortness of breath (28, 37), palpitations (28), and limb edema (28). The use of telemedicine also enhanced the timeliness of respiratory infection diagnosis (37). Batalik et al. reported that telerehabilitation improved cardiac and respiratory functions and reduced walking test times in patients with coronary artery diseases (16). Teleconsultation also aided cardiac rehabilitation for patients with acute myocardial infarction (31). Hospitalization rates were reduced using telemedicine services compared to in-person visits (7, 15, 26, 33).

Moreover, patient consultations with physicians increased through telemedicine (28, 30, 32, 34). Televisits were more effective than in-person visits (31), and teleclinic services decreased heart transplant surgeries for patients with heart failure (34). Overall, telemedicine improved physical activity, mental wellness, anxiety reduction, nutritional well-being, prevention of weight gain, and immunity to Covid-19 (25, 27, 37). It also facilitated medication dosage control (34), medication adherence (31), smoking cessation (31), follow-up quality (35), and health status monitoring (36) for patients with cardiovascular diseases.

#### 3.4.2 Non-clinical impact

Several studies demonstrated that the implementation of teleconsultation services has resulted in increasing satisfaction among physicians, nurses (31, 33), and patients (33). In addition, teleconsultation services can efficiently decrease waiting times (29), and the time required for in-person visits (7, 18, 31). Some studies showed that using telemedicine services during the Covid-19 pandemic had some advantages over in-person consultations. These included reducing the workload of physicians (29, 31), saving travel costs (31), preventing readmissions (26, 34), and improving healthcare quality (16).

### 3.5 Synthesis

Overall, the results of this study indicated that telephone consultation (*n* = 9, 53%) and videoconferencing (*n* = 7, 41.1%) were the most common tools for providing telemedicine services. CardioClick, Heartbeat Health, and Zoom were the most commonly used platforms for videoconferencing. The findings also indicated that the primary non-clinical impacts of telemedicine services for patients with cardiovascular diseases during the Covid-19 pandemic included the reduction of in-person visits and healthcare providers' workload. The clinical impacts included decreasing re-admission rates, improving vital signs, and quality of care.

## 4 Discussion

The present study examined the applications of telemedicine for cardiovascular diseases management during the Covid-19 pandemic. According to the results, teleconsultations and televisits were the most used services and the main tools were telephone and videoconferencing. The use of telemedicine had some clinical and non-clinical impacts which could improve quality of care and cardiac rehabilitation, and reduce waiting times and healthcare costs.

Similar to the findings of this study, other studies have demonstrated that offering teleconsultation services, particularly for cardiovascular patients, has resulted in reducing the risk of Covid-19 infection (38). Furthermore, several studies showed that teleconsultation services have significantly improved clinical symptoms compared to the in-person consulattions (39, 40). This was an effective approach for reducing in-person visits, readmissions, and referrals. Better medication adherence, improving clinical symptoms, and easier patient follow-up during the Covid-19 pandemic were among other benefits of teleconsultations.

The results showed that televisit services during Covid-19 pandemic had a number of benefits, such as reducing the physician's workload and saving their time. Similarly, Gorodeski et al. showed that televisit services might be helpful for patients with heart failure to reduce re-hospitalization rates and to improve clinical outcomes (41). Brunettiet al. showed that using televisits and electrocardiograms reduced the duration of emergency care for patients with cardiovascular diseases (19, 42).

Regarding the technology and communication tools, telephone and videoconferencing were the most commonly used technologies for the provision of telemedicine services to the patients with cardiovascular diseases during the Covid-19 pandemic. The results of several studies showed that the use of telephone consultation was effective in providing ongoing support for maintaining and promoting behavioral health changes, medication adherence, and controlling risk factors for cardiovascular diseases (43, 44).

Moreover, telephone counseling can effectively reduce healthcare costs for cardiovascular patients (42). This type of services led to a reduction in hospital length of stay, number of referrals, and number of hospitalizations, surgeries, and emergency surgeries during the Covid-19 era (43). Similarly, the use of videoconferencing led to diagnose symptoms and treat patients as early as possible, reduce re-hospitalizations, decrease emergency care visits, and save healthcare costs (42, 44, 45). Idris et al. showed that videoconferencing can be effective for patients with heart failure in scheduling weekly visits (46).

The use of mobile-based applications was another approach to manage cardiovascular diseases. For instance, Sua et al. demonstrated that self-management interventions using mobile-based applications were correlated with a decrease in blood pressure among individuals with cardiovascular conditions (47). Similarly, Rawstorn et al. found that telemedicine and telecardiac rehabilitation were effective in controlling diastolic blood pressure, reducing low-density lipoprotein cholesterol, and improving respiratory capacity (48).

However, the findings of Huang et al.'s research indicated that the implementation of telemedicine interventions did not yield a statistically significant correlation with enhanced cardiac rehabilitation for patients. Furthermore, it failed to induce improvements in clinical symptoms such as weight loss, blood pressure, cholesterol levels, smoking cessation, and quality of life (49).

The findings demonstrated that telemedicine and mobile phone applications were effective in reducing the hospitalization rates of patients with cardiovascular diseases during the Covid-19 pandemic. Similarly, several studies showed that telemedicine interventions effectively decreased the rate of re-admission among patients with cardiovascular diseases compared to the standard care (50–54). In contrast, some researchers reported that the implementation of telemonitoring did not have an impact on the re-admission rate of patients with heart failure (55, 56).

Improving clinical care, quality of life, and psychological well-being for patients with heart failure were other benefits of using telemedicine services during the Covid-19 pandemic. Marra et al. found that using telephone consultation and videoconferencing services was significantly effective in improving nutritional well-being in patients with cardiovascular diseases (57).

Several studies reported that using telemedicine in the management of patients with heart failure has resulted in a reduction in mortality rate (52, 58–60). Similarly, the study conducted by Gallagher et al. revealed that providing post-discharge telephone consultations can improve treatment adherence in patients with heart failure (44, 61). The results of several studies demonstrated that telephone consultation is especially helpful for the early diagnosis of cardiovascular diseases and reduces diagnostic costs (42, 52, 62). Van Dyck et al. found that using telemonitoring services decreased the number of emergency department visits and reduced 15% healthcare costs (63). Other studies reported that using telemedicine significantly improves physical activity and quality of life in patients with coronary heart disease (59, 64).

It is notable that during the Covid-19 pandemic, managing heart failure in patients with dilated cardiomyopathy (DCM) posed significant challenges that were effectively mitigated through telemedicine technologies. DCM is characterized by the heart's diminished ability to pump blood effectively, and requires continuous and comprehensive management to prevent exacerbations and hospitalizations (65). Telemedicine enabled remote management by providing tools for regular teleconsultations, which allowed healthcare providers to assess DCM symptoms, adjust treatments, and offer immediate medical advice without necessitating in-person visits (66, 67).

Telemonitoring technologies, such as wearable devices and mobile health applications, played a crucial role by continuously tracking vital signs like blood pressure, heart rate, and weight (68). These technologies facilitated early detection of worsening conditions, prompting timely medical interventions for DCM patients that prevented hospital admissions. In addition, virtual cardiac rehabilitation programs supported DCM patients in maintaining their physical activity and managing their condition during lockdowns (69, 70).

Studies showed that these telehealth interventions led to reduced hospital readmissions, improved medication adherence, and better management of heart failure symptoms. The ability to provide real-time, patient-specific care remotely proved invaluable in maintaining the health and safety of DCM patients, minimizing their exposure to Covid-19, and ensuring continuous, effective disease management (32). This approach not only addressed the immediate needs during the pandemic but also highlighted the potential for ongoing telemedicine integration in managing chronic cardiovascular diseases (71).

### 4.1 Limitations

This study had some limitations. The first limitation was related to the number of databases and the inclusion criteria set for the selecting the articles. In this study, certain databases were searched and only studies published in English were included. There might be other articles indexed in different databases and published in non-English languages that were not included in this study. However, the researchers believed that the main databases were searched in this study to include as many relevant articles as possible. The second limitation might be related to the type of the study which was a scoping review. Although this study helped to provide an overview of the applications of telemedicine for cardiovascular diseases management during the Covid-19 pandemic, the impact of this intervention was not measured quantitatively. Future research can focus on conducting systematic reviews and meta-analysis to identify the clinical and non-clinical impact of telemedicine using quantitative approaches.

## 5 Conclusion

This study investigated the application of telemedicine for cardiovascular diseases management during the Covid-19 pandemic. The findings showed the efficacy of using telemedicine for cardiovascular diseases management, especially in terms of clinical and non-clinical impacts. The results suggested the potentials of telemedicine technology for managing patients with cardiovascular diseases even in the post-Covid-19 era. Future research should concentrate on identifying practical strategies for using telemedicine in cardiovascular diseases management, considering patients' and healthcare providers' perspectives. In addition, future studies should investigate new solutions to improve the accessibility, affordability, and privacy of telemedicine services.

## Appendix

Appendix I: Search strategies

**Table:**

No.	Database	Search strategy
1	PubMed	(Telemedicine [Title/Abstract] OR Telemedicine [MeSH Terms] OR ehealth [Title/Abstract] OR mhealth [Title/Abstract] OR Telehealth [Title/Abstract] OR teleconsultation [Title/Abstract] OR telerehabilitation [Title/Abstract] OR “Remote Consultation”[Title/Abstract]) AND (“Cardiovascular diseases”[Title/Abstract] OR “cardiovascular diseases” [MeSH Terms] OR “Coronary heart disease”[Title/Abstract] OR “Peripheral arterial disease”[Title/Abstract] OR “rheumatic heart disease”[Title/Abstract] OR “congenital heart disease”[Title/Abstract] OR “Deep vein thrombosis”[Title/Abstract]) AND (Covid-19 [Title/Abstract] OR coronavirus [Title/Abstract] OR “SARS-COV-2”[Title/Abstract] OR “Sever acute respiratory syndrome coronavirus”[Title/Abstract])
2	Web of Science	TS = ((“Telemedicine” OR “ehealth” OR “mhealth” OR “Telehealth” OR “telerehabilitation”) AND (“Cardiovascular diseases” OR “Coronary heart disease” OR “Peripheral arterial disease” OR “rheumatic heart disease” OR “congenital heart disease” OR “Deep vein thrombosis”) AND (“Covid-19” OR “coronavirus” OR “SARS-COV-2” OR “Sever acute respiratory syndrome coronavirus”))))
3	Scopus	TITLE-ABS-KEY((“Telemedicine” OR “ehealth” OR “mhealth” OR “Telehealth” OR “telerehabilitation”) AND (“Cardiovascular diseases” OR “Coronary heart disease” OR “Peripheral arterial disease” OR “rheumatic heart disease” OR “congenital heart disease” OR “Deep vein thrombosis”) AND (“Covid-19” OR “coronavirus” OR “SARS-COV-2” OR “Sever acute respiratory syndrome coronavirus”))
4	The Cochrane Library	(“Telemedicine” OR “ehealth” OR “mhealth” OR “Telehealth” OR “telerehabilitation”) AND (“Cardiovascular diseases” OR “Coronary heart disease” OR “Peripheral arterial disease” OR “rheumatic heart disease” OR “congenital heart disease” OR “Deep vein thrombosis”) AND (“Covid-19” OR “coronavirus” OR “SARS-COV-2” OR “Sever acute respiratory syndrome coronavirus”) in Title Abstract Keyword
5	Ovid Medline	((“Telemedicine” OR “ehealth” OR “mhealth” OR “Telehealth” OR “telerehabilitation”) AND (“Cardiovascular diseases” OR “Coronary heart disease” OR “Peripheral arterial disease” OR “rheumatic heart disease” OR “congenital heart disease” OR “Deep vein thrombosis”) AND (“Covid-19” OR “coronavirus” OR “SARS-COV-2” OR “Sever acute respiratory syndrome coronavirus”)).ti. or ((“Telemedicine” OR “ehealth” OR “mhealth” OR “Telehealth” OR “telerehabilitation”) AND (“Cardiovascular diseases” OR “Coronary heart disease” OR “Peripheral arterial disease” OR “rheumatic heart disease” OR “congenital heart disease” OR “Deep vein thrombosis”) AND (“Covid-19” OR “coronavirus” OR “SARS-COV-2” OR “Sever acute respiratory syndrome coronavirus”)).ab. or ((“Telemedicine” OR “ehealth” OR “mhealth” OR “Telehealth” OR “telerehabilitation”) AND (“Cardiovascular diseases” OR “Coronary heart disease” OR “Peripheral arterial disease” OR “rheumatic heart disease” OR “congenital heart disease” OR “Deep vein thrombosis”) AND (“Covid-19” OR “coronavirus” OR “SARS-COV-2” OR “Sever acute respiratory syndrome coronavirus”)).kw.
6	IEEE Xplore	(((“Telemedicine” OR “ehealth” OR “mhealth” OR “Telehealth” OR “teleconsultation” OR “telerehabilitation” OR “Remote Consultation”) AND (“Cardiovascular diseases” OR “Coronary heart disease” OR “Peripheral arterial disease” OR “rheumatic heart disease” OR “congenital heart disease” OR “Deep vein thrombosis”) AND (“Covid-19” OR “coronavirus” OR “SARS-COV-2” OR “Sever acute respiratory syndrome coronavirus”)))
7	CINAHL	(“Telemedicine” OR “ehealth” OR “mhealth” OR “Telehealth” OR “telerehabilitation”) in Title Abstract Keyword AND (“Cardiovascular diseases” OR “Coronary heart disease” OR “Peripheral arterial disease” OR “rheumatic heart disease” OR “congenital heart disease” OR “Deep vein thrombosis”) in Title Abstract Keyword AND (“Covid-19” OR “coronavirus” OR “SARS-COV-2” OR “Sever acute respiratory syndrome coronavirus”) in Title Abstract Keyword -(Word variations have been searched)
8	ProQuest	title((“Telemedicine” OR “ehealth” OR “mhealth” OR “Telehealth” OR “telerehabilitation”) AND (“Cardiovascular diseases” OR “Coronary heart disease” OR “Peripheral arterial disease” OR “rheumatic heart disease” OR “congenital heart disease” OR “Deep vein thrombosis”) AND (“Covid-19” OR “coronavirus” OR “SARS-COV-2” OR “Sever acute respiratory syndrome coronavirus”)) OR abstract (((“Telemedicine” OR “ehealth” OR “mhealth” OR “Telehealth” OR “telerehabilitation”) AND (“Cardiovascular diseases” OR “Coronary heart disease” OR “Peripheral arterial disease” OR “rheumatic heart disease” OR “congenital heart disease” OR “Deep vein thrombosis”) AND (“Covid-19” OR “coronavirus” OR “SARS-COV-2” OR “Sever acute respiratory syndrome coronavirus”)) OR mainsubjects ((“Telemedicine” OR “ehealth” OR “mhealth” OR “Telehealth” OR “telerehabilitation”) AND (“Cardiovascular diseases” OR “Coronary heart disease” OR “Peripheral arterial disease” OR “rheumatic heart disease” OR “congenital heart disease” OR “Deep vein thrombosis”) AND (“Covid-19” OR “coronavirus” OR “SARS-COV-2” OR “Sever acute respiratory syndrome coronavirus”))
9	Google Scholar	allintitle: (“Telemedicine” OR “ehealth” OR “mhealth” OR “Telehealth” OR “telerehabilitation”) AND (“Cardiovascular diseases” OR “Coronary heart disease” OR “Peripheral arterial disease” OR “rheumatic heart disease” OR “congenital heart disease” OR “Deep vein thrombosis”) AND (“Covid-19” OR “coronavirus” OR “SARS-COV-2” OR “Sever acute respiratory syndrome coronavirus”)
